# The Extracellular Matrix Contributes to Mechanotransduction in Uterine Fibroids

**DOI:** 10.1155/2014/783289

**Published:** 2014-07-03

**Authors:** Phyllis C. Leppert, Friederike L. Jayes, James H. Segars

**Affiliations:** ^1^Duke University School of Medicine, Durham, NC 27710, USA; ^2^Unit on Reproductive Endocrinology and Infertility, Program on Pediatric and Adult Endocrinology, NICHD, NIH, Bethesda, MD 20892-1109, USA

## Abstract

The role of the extracellular matrix (ECM) and mechanotransduction as an important signaling factor in the human uterus is just beginning to be appreciated. The ECM is not only the substance that surrounds cells, but ECM stiffness will either compress cells or stretch them resulting in signals converted into chemical changes within the cell, depending on the amount of collagen, cross-linking, and hydration, as well as other ECM components. In this review we present evidence that the stiffness of fibroid tissue has a direct effect on the growth of the tumor through the induction of fibrosis. Fibrosis has two characteristics: (1) resistance to apoptosis leading to the persistence of cells and (2) secretion of collagen and other components of the ECM such a proteoglycans by those cells leading to abundant disposition of highly cross-linked, disoriented, and often widely dispersed collagen fibrils. Fibrosis affects cell growth by mechanotransduction, the dynamic signaling system whereby mechanical forces initiate chemical signaling in cells. Data indicate that the structurally disordered and abnormally formed ECM of uterine fibroids contributes to fibroid formation and growth. An appreciation of the critical role of ECM stiffness to fibroid growth may lead to new strategies for treatment of this common disease.

## 1. Introduction

Uterine fibroids are firm, stiff nodular tumors, a fact understood by all clinicians and confirmed by biomechanical studies (as discussed in [1–3]). The proteins of the extracellular matrix (ECM), particularly the interstitial collagens are responsible for this property of “firmness” and for the mechanical strength of the tissue. In biomechanical terms this property is referred to as stiffness. Fibroids feature an accumulation of altered collagen and differing amounts of glycosaminoglycans along with proliferation of cells, which is by definition fibrosis. A complete understanding of the role of the ECM proteins, especially collagen, and their effect on growth and development of fibroids must take into account the process of mechanotransduction, a dynamic system whereby mechanical forces initiate chemical signaling within cells. This bidirectional process is promoted by the stiffness of the ECM and initiates cell-matrix interactions in a progressive, ever changing process leading to activation of signaling cascades. While soluble signaling molecules have long been recognized as important factors in fibroid growth, development, differentiation, and function, mechanical signaling has only more recently been shown to play a critical role.

Here we focus on the ECM and the properties of cell and tissue* stiffness *that lead to signal transduction and then review current knowledge regarding the ECM of uterine fibroids. This information is placed in the context of the modern appreciation of how the ECM functions in other tissues and organs and the contemporary understanding of mechanotransduction. It is universally accepted that a myriad of chemical signaling molecules has been identified in fibroids and that reproductive tract hormones regulate the complex systems leading to fibroid development and growth. These chemical and hormonal signals are part of the biological mechanism of the development of these tumors, but these signals alone do not fully account for fibroid development and growth. We provide evidence in this review to suggest that mechanical force contributes to the triggering of these signaling pathways and plays a major role in the pathobiology of uterine fibroids.

## 2. Mechanotransduction: A Mechanism of Disease

**Figure 1:**
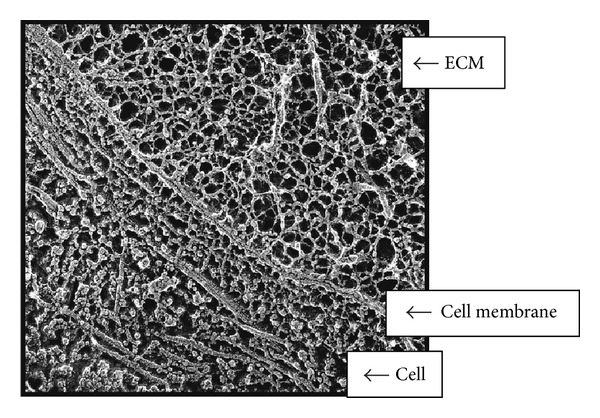
Interconnected structural components of cells and ECM. Quick freeze, deep etch electron micrograph of a fetal ear cartilage chondrocyte cell to illustrate the integration of structures inside and outside the cell. The ECM (containing a meshwork of proteoglycans, collagen, fibronectin, and laminin), the cell membrane (integrins receptors), and the cell (containing microtubules) are visualized. In the ECM the thinnest fibrils in the meshwork are 4 ± *I* nm and are presumed to be proteoglycans. The larger thicker fibrils are collagen. Micrograph courtesy of Robert Mecham and John Heuser, Washington University, St. Louis, MO, USA. This figure illustrates that mechanical forces can be transmitted across the cell surface and into the cell by means of interconnected structural components (as discussed in [4]).

Individual cells are capable of translating mechanical stimuli such as stretch or compression from their environment into biochemical signals through a complex system of cytoskeletal ECM receptors and transmembrane molecules that interconnect with integrin subunits and cell surface proteoglycans (as discussed in [4]). The highly dynamic mechanotransduction process is defined as cooperative signaling between the cell and the ECM. It has been suggested that this mechanotransduction system would be able to transfer signals through the entire cell and therefore would be more rapid than the diffusion based system (discussed in [4]). In response to mechanical force in living tissue there is a reciprocal or “back and forth” activity where extracellular remodeling, rapid transient cell-cell, and cell-matrix interactions all have essential functions (as discussed in [5, 6]). This interaction occurs between one cell and its extracellular matrix as well as between many cells and the matrix within tissues. Mechanical force is an upstream signal and will initiate physiological processes such as those in development and disease states. Recent studies show that ECM collagen provides a dynamic role in the breast cell microenvironment and is actually active in the promotion of tumor progression (as discussed by [7]). Increased ECM stiffness generated by collagen regulates breast epithelial signaling through integrin activation of *β*-catenin and MYC (a transcription factor persistently expressed in cancers, so named because it is similar to the myelocytomatosis viral oncogene) to activate the microRNA-18a expression that drives breast tumor progression (as discussed by [8]). This tumor progression is induced by MiRna reduction of tumor suppressor phosphatase and tensin homolog (PTEN) by the reduction of homoebox A9 (HOXA9) (as discussed by [8]). Mechanotransduction can be altered by mutations in downstream signaling cascades. Abnormal mechanical signaling as a result of modifications in these interactions or cellular sensitivity to mechanical stress contributes to disease development (as discussed in [6]). The molecules in the cell-ECM network appear to be linked mechanically from the ECM to the cytoskeleton (Figure 1). Intriguingly, recent work has indicated a system linked from the ECM through the cytoskeleton to the nucleus (as reported in [9]).

While the cell's membrane receptors, especially integrins, allow the transmission of mechanical signals across the cell surface, change in the ECM structure and the amount within the microenvironment are the sources of the mechanical forces. This dynamic relationship is noted in the disease process of fibrosis. The formation of fibrosis correlates with new collagen deposition and TGF-beta-induced myofibroblast differentiation, as well as elastin gene expression, WNT-1 inducible signaling pathway protein-1, lysyl oxidase, and type V collagen gene expression (as discussed in [10]). In addition there is often the accumulation of glycosaminoglycans as well as resistance to apoptosis in fibrotic tissue (as discussed in [11] and references therein). Fibrosis occurs when there is a hyperproduction of collagen and lack of appropriate remodeling and degradation. The increased accumulation of the ECM observed in fibrotic tissue causes stiffness in the microenvironment of the individual cells in tissues and organs and contributes to mechanotransduction (as discussed in [4, 8]). While the individual cell contributes in this dynamic process and changes within it cause signals to the ECM, this review is focused on the ECM and its functions. As the ECM accumulates additional mechanical force is produced by the ECM. To complete the picture, the role of extracellular fluid (ECF) in the generation of intrinsic mechanical force is now accepted (as discussed in [11]). Conformational changes in proteins that are mechanosensitive are sensed by the cells and open membrane channels, alter binding affinities, and increase phosphorylation thus activating downstream signaling pathways. Although the specific pathways of mechanotransduction have not been completely elucidated in uterine myometrial or fibroid cells, it is reasonable to suggest that mechanical signaling pathways exist in these cells as they do in the cells of tissues studied so far.

**Figure 2:**
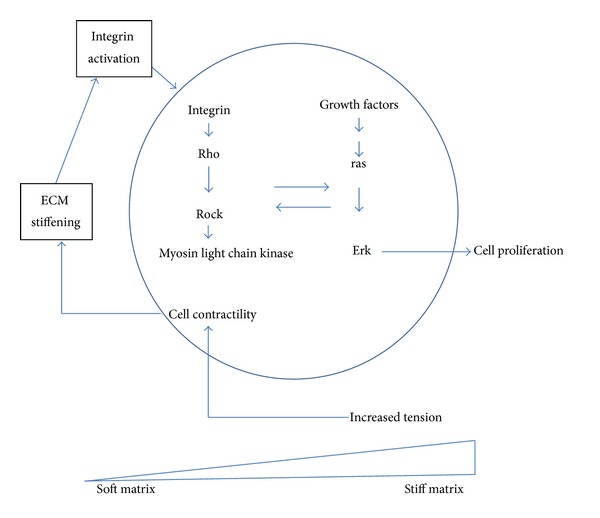
Elements of mechanical signaling. A simplified pathway of mechanical signaling in cells is depicted. As the cell cytoskeleton contracts (a process called cell contractility) and ECM accumulates in the cell microenvironment, integrin activation occurs leading to activation of Rho (as discussed in [1]). Rho in turn activates ROCK leading to activation of ras. In fibroid cells obtained either at the time of hysterectomy or myomectomy, RhoA activity is attenuated. This adaptation of the fibroid cell is not ROCK dependent (as discussed in [2]). These findings suggest that fibroid cells in symptomatic tumors where treatment was needed are fundamentally adapted to their stiff microenvironment and thus become insensitive to moderate mechanical cues. It is not certain when in the natural history of fibroid development that this adaptive response to mechanotransduction occurs. Nevertheless, mechanical sensing does occur in fibroid cells.

The ECM is able to initiate the mechanotransduction process and actually serves as a reservoir for matricellular proteins, growth factors, and cytokines. Often, activation of these factors occurs in the ECM. For example, TGF is activated in the ECM by a very complex mechanism. The ECM is able to transmit physical and chemical signals to the cell membrane leading to signal propagation and amplification via a multiplex array of intracellular signals. Specifically, the Rho/ROCK/MAPK signaling pathway is one important pathway in mechanotransduction (Figure 2). RhoA belongs to a family of small GTP-binding proteins. Both RhoA and Rho-kinase (ROCK), a downstream target of Rho, are involved in the regulation of a wide variety of cellular processes, including changes in cell motility and morphology, focal adhesion formation, and light chain phosphorylation (as discussed in [12]). An important concept is that reciprocity is an indispensable quality of mechanosensitive cells. Cells respond to biophysical and biochemical cues from the ECM, but cells also help maintain and remodel the ECM through secretion of various ECM components and the molecules that degrade the ECM. Thus, ECM remodeling will produce a matrix that can be either flexible or stiff depending on protein cross-links, content of glycoproteins, hydration, and other components of the ECM.

Mechanotransduction has long been viewed as a mechanism of disease (as discussed in [13]).

Mechanical signaling has been well described in dermal wounds and has also been studied in other tissues such as blood vessels, stem cells, respiratory epithelium, and musculoskeletal tissues. Notably, the reproductive system is dynamic and constantly undergoes extensive structural remodeling throughout each reproductive cycle—featuring a remarkable plasticity of the ECM. This remodeling is achieved, in part, by changes in the ECM, which are regulated by cyclic hormonal cues as well as cytokines and growth factors. Mechanotransduction is highly reciprocal and involves extensive bidirectional signals from the ECM to and from the cell (as presented and discussed in [14]).

## 3. Extracellular Matrix in the Human Uterus

Appreciation of the complexities of the ECM is essential to the understanding of the fibrotic process and its role in mechanotransduction in the human uterus. The uterine myometrium consists mainly of smooth muscle cells that stain positive for *α* smooth muscle actin and desmin and are interspersed with interstitial collagens. These collagens are large rigid molecules that are responsible for the mechanical strength of uterine tissue as well as all other tissues in the body. Histological examination demonstrates a basketweave pattern of the smooth muscle cells and the interstitial collagens (as discussed in [15, 16]). Notably, myometrium is a complex tissue and heterogeneous in the proportion of tissue stained by Masson Trichrome, a collagen stain (as discussed in [17]). Trichrome staining of tissue, however, will not differentiate between the numerous types of human interstitial collagens. At the ultrastructural level the interstitial collagen fibrils are observed in parallel to the smooth muscle cells, a feature which allows the uterus to maintain its strength as it expands during pregnancy (as discussed in [18]). The collagen superfamily of proteins is complex in molecular organization and is distributed widely in tissues and diverse in function (as discussed by [19]). Twenty-nine types of collagens encoded by numerous genes located on different chromosomes are known in humans.

The predominate collagens found in the interstitial areas of the myometrium are Type I, Type III, and Type V, although types IV and VI are also present in the uterus (as discussed in [20–24]). Type IV collagen is membrane collagen, arrayed as a meshwork (as discussed in [25]). All collagens have three polypeptide chains and have tandem repeated Gly-Xaa-Yaa sequences, where X is proline and Y is hydroxyproline. These peptide chains are able to assemble into stable triple helical structures. Glycine is the smallest amino acid and its location in the collagen molecule allows for a tightly coiled helix as glycine is easily “packed” into the center of the assembled helix. The hydroxyprolines are located on the outside of the triple helix stabilizing the molecule. Three alpha chains make up a collagen molecule. As examples, Type I, the most abundant collagen type in the human body is made up of chains encoded by the genes COL1A1 and COL1A2. COL1A1 gene produces the pro-alpha 1 (I) chain and the COL1A2 gene produces the pro-alpha 2(I) chain. Two pro-alpha 1 chains and one pro-alpha 2 chain form type 1 procollagen. Type III collagen consists of three alpha 1(III) chains encoded by COL3A1. Type IV collagen consists of two alpha 1 IV chains and one alpha 2 IV chain and is the type of collagen found in membranes, blood vessels, and nerves (as discussed in [26]).

The biosynthesis of collagen is complicated. Therefore, gene expression studies alone do not reflect the abundance of native, cross-linked collagen in tissue. In general, collagen synthesis includes extensive co- and posttranslational modifications that stabilize the helix and assist with higher order molecular assembly (shown below) (as discussed in [26]).

*Synthesis of Type I Collagen: An Interstitial Collagen.*

1. COL1A1 gene →pro-alpha1 (I) chain,
2. COL1A2 gene → pro-alpha2 (I) chain,
3. in ER → preprocollagen,
4. ER Lumen → signal peptides cleaved; proline and lysine hydroxylation,  2 pro-alpha1 and 1 pro-alpha2 chains → Type I procollagen, triple helix formed →
5. in Golgi packaged and secreted →
6. outside cell propeptides cleaved → tropocollagen
7. fibrils formed,
8. lysyl oxidase creates intramolecular and intermolecular cross-links.

In the above list synthesis of Type I collagen is described. Other interstitial collagens, such as Type III and Type V, are synthesized in a similar manner.

Briefly, the genes are transcribed and their mRNAs are processed similarly to other molecules. Then the peptide chains are translated in the rough endoplasmic reticulum (RER). These peptide chains are known as preprocollagen and have a signal peptide and propeptides on each end. The signal peptide is cleaved to form pro-alpha chains. In the lumen of the RER, hydroxylation of the proline and lysine amino acids occurs, a step dependent on ascorbic acid (vitamin C) (as discussed in [26]). Specific hydroxylysine residues are glycosylated and the triple helical structure as noted is formed from two alpha-1 chains and one alpha-2 chain. This assembly is assisted by disulfide bonding between the chains (as discussed in [27]). The procollagen molecule has an N-terminal propeptide, the central helix, and a C-propeptide region (as discussed in [28]). This tight triple helix ensures that this part of the molecule is inaccessible to modifying enzymes and the propeptides prevent fibril formation inside the cell. Next, the procollagen moves to the Golgi where it is packaged and secreted (as discussed in [26]).

Once outside the cell, the collagen propeptides are cleaved in the extracellular space and tropocollagen is formed by the enzymatic action of procollagen peptidase (as discussed in [29]). In the case of Types I and III collagens, the tropocollagen molecules spontaneously form fibrils by the alignment of charged and hydrophobic amino acid clusters (as discussed in [30]). These fibrils are laid down in a staggered fashion such that a d-band of alternating regions of protein density in the fibril produces a characteristic gap and overlap appearance of negatively contrasted fibrils observed in transmission electron microscopy (as discussed in [31]). Finally in the extracellular space copper-dependent lysyl oxidase (LOX) enzymatically creates the intramolecular and intermolecular cross-links that form the mature collagen fibrils (as discussed in [32, 33]). These covalent cross-links are di-, tri-, and tetrafunctional and further stabilize the collagen helix (as discussed in [34]). The more lysine derived cross-links in collagenous tissue, the stiffer the tissue. In some tissues Type I and Type III collagen molecules are present within the same collagen fibril, covalently bound between the N-terminal regions (as discussed in [35]). In liver fibrosis Types I and III collagens colocalize in collagen fibers (as discussed in [36]) and while this has not been studied in the uterine fibrosis this type of fiber could be a possibility in that situation. However, in most tissues the collagen fibrils consist of all Type I collagen molecules or all Type III collagen molecules with tissues characterized by specific Type I/Type III ratios. In pathological situations cells are capable of altering this ratio. For instance, in healing wounds there is an increase in Type III collagen and in some situations an increase in Type V collagen as well.

Other major components of the uterine ECM are proteoglycans which are glycoproteins that contain a core protein with covalently attached high negatively charged glycosaminoglycan (GAG) side chains. GAGs are sulfated polysaccharides that contain repeating disaccharides (as discussed in [26]). There is one exception and that is hyaluronic acid. It is not attached to a protein core and it is not sulfated (as discussed in [26, 37]). The disaccharides are unique to each proteoglycan. Glucuronic acid-N-acetylgalactosamines are components of chondroitin sulfate; iduronic acid-N-acetylgalactosamines are units of dermatan sulfate; heparin contains glucuronic acid-N-acetylglucosamines and heparan sulfate contains glucuronic acid-N-acetylglucosamines. Keratan sulfate is the one glycosaminoglycan that does not contain uronic acid; it contains N-acetylglucosamine-galactose (as discussed in [26]). Proteoglycan synthesis begins with the core protein which is then modified in the RER. After this monosaccharides and sulfate, the building blocks for the GAGs are taken up by the cell through specialized transporters in the plasma membrane (as discussed in [36]). These molecules are activated in the cytosol to form UDP-sugars and 5′-phosphosulfate (PAPS) and transported to the Golgi (as discussed in [37]). In the Golgi, the monosaccharides (xylosyl-, galactosyl-galactosyl-uronic acid) and the linker tetrasaccharide are attached to the core protein at selected serine residues, and additional disaccharides are attached to the proteoglycan. The addition of a fifth saccharide then determines if the GAG chain will become chondroitin sulfate, dermatan sulfate, or heparan sulfate or heparin (as discussed in [37]). Heparan sulfate and heparin undergo even more complex modification. When polymerization is finished, O-sulfation occurs. This modification is different depending on the proteoglycan (as discussed in [37]). Proteoglycans attract cations and bind water enabling tissues to accommodate to pressure changes. Proteoglycans also play important roles in the control of cell growth and differentiation. The small dermatan sulfate proteoglycan decorin, a member of the small leucine-rich repeat class of proteins (SLRP) through its interaction with collagen, may aid in the adaption of tissue to increased mechanical loads (force) (as discussed in [38]). Decorin interacts with specific regions of the collagen fibrils and regulates their formation (as discussed by [39–42]). Decorin and collagen colocalize in both myometrium and fibroids indicating this interaction may be present in the uterus (as discussed in [43]). Larger and longer GAGs of the side chains on the decorin molecule most likely exert higher osmotic pressure and thus affect both ECM organization and matrix-cell interaction. Decorin has been studied in the uterine cervix and appears to have a role in the cervical changes of gestation and parturition by interaction with collagen fibers (as discussed by [44–46]). Hyaluronan becomes dominant at term cervix in mice and women and contributes to the loss of tensile strength of the cervix during parturition (as discussed in [47, 48]). Hyalectans, another family of ECM proteoglycans, interact with hyaluronic acid and are very important in the regulation of water retention and distribution in tissue. One member of this family is versican, a protein glycosylated with galactosaminoglycan side chains and which is increased in tumors compared to normal tissue (as discussed in [49, 50]).

In addition to collagen and glycosaminoglycans, elastin, a hydrophobic ECM protein is an important component of the uterus. Elastin is responsible for the ability of tissue to stretch and recoil and is a hydrophobic protein with a complex synthesis (as discussed in [51]). Elastin is found in the uterus in fibrils and thin sheets from 0.1 to 0.4 microns in thickness that are capable of multidimensional stretch, in contrast to the elastin lamellae found in the aorta which are 1 to 2.5 microns in thickness and stretch in one dimension (as discussed in [52]). The uterine elastin is so thin that elastic fibers are often not seen in histological sections of nonpathological conditions (as discussed in [52]). During pregnancy, elastin allows the uterus to achieve its remarkable increase in size, to stretch, and to accommodate the growing fetus.

Mechanical force is transmitted across the cell membrane by integrins, transmembrane receptors expressed in all cells. These surface molecules are important in both cell to matrix (inside out) and ECM to cell signaling (outside in) and act as mechanosensors generating signals that affect cell physiology and pathology by complex mechanisms (as discussed in [53, 54]). Integrins are heterodimers consisting of an alpha subunit and a beta subunit and are involved in numerous processes such as adhesion, ECM organization, signaling, cell survival, proliferation, and in mechanotransduction (as discussed in [55]). Although there are multiple integrins that bind to many proteins, only those pertinent to this review are mentioned here. The references cited discuss many additional integrins and their functions. There are four collagen-binding integrins in the *β*1 subfamily, namely, *α*1*β*1, *α*2*β*1, *α*10*β*1, and *α*11*β*1 (as discussed in [53]). Three of these collagen binding integrins have been demonstrated to be present in fibroids as well as in the myometrium and in the cervix (as discussed in [56–59]). Integrins binding to fibronectin, *α*3*β*1, *α*4*β*1, *α*5*β*1, *α*v*β*1, and *α*v*β*3 and to laminin, *α*1*β*1, *α*3*β*1, *α*6*β*1, and *α*v*β*3 are also expressed in myometrium (as described by [57]). The integrin subunit *β*1 is expressed in myometrium and in uterine fibroids (as discussed in [57, 60]). Fibronectin, an ECM molecule with multiple functions mediates the assembly of the matrix binding collagen, integrins, and other ECM molecules by undergoing a series of conformational changes that expose cryptic binding sites (as discussed in [61]). Mechanical forces are capable of inducing further conformational changes in fibronectin thus mediating multiple effects on ECM and cell and tissue functions (as discussed in [62]). Thus fibronectin is an organizer of matrix assembly (as discussed by [53, 60]).

The turnover and degradation of the ECM is also complex. Humans are considered to have 23, or as some suggest, up to 28 matrix metalloproteinases (MMPs) that degrade both matrix and nonmatrix proteins and are important in tissue repair and remodeling (as discussed in [63–65]). These enzymes break down cell-surface and ECM molecules that alter cell-matrix or cell-cell interactions and also release growth factors not related to the degradation of collagen or other matrix molecules including proteoglycans (as discussed in [64]). MMPs play roles also in cell migration, growth, differentiation, apoptosis, and inflammatory responses (as discussed in [65]). All MMPs utilize Zn^2+^ ion linked to their catalytic site to hydrolyze specific peptide substrates. After secretion MMPs must be activated by protein cleavage. For example, ProMMP-1 is activated by cleavage of its propeptide by MMP-3 (as discussed in [66]). MMP-1 binds to and locally unwinds the triple helix before it hydrolyses the collagen molecule into 2 fragments (as discussed in [67]). MMPs are inhibited by tissue inhibitors of matrix metalloproteinases (TIMPs) and *α*_2_-macroglobulin. Thus MMPs are regulated in several ways: during gene expression, by the need for activation of latent proenzymes and by being bound to inhibitory molecules. This equilibrium between activation and inhibition is delicate and results in situations where a particular signal may regulate an MMP in the direction of activation but will regulate another MMP by inhibition (as discussed by [68]). The regulation of TIMPs in some situations can be hormone-dependent (as discussed by [68]). Demonstration of the presence of MMP mRNA or protein in a particular tissue, however, does not prove how MMP is functioning in that tissue (as discussed by [64, 68]). Recent compelling studies demonstrate that vitamin D inhibits the nuclear factor-*κ* *β* (NF-*κ* *β*) pathway directly regulating genes that contribute to cell proliferation, inflammation, fibrogenesis, increased oxidative stress, and decreased MMP-9 (as discussed by [69]). Experiments in rats showed that vitamin D reduced ECM deposition in induced liver fibrosis and lowered the fibrotic score in the animals (as discussed by [70]).

## 4. Extracellular Matrix of Uterine Fibroids

**Figure 3:**
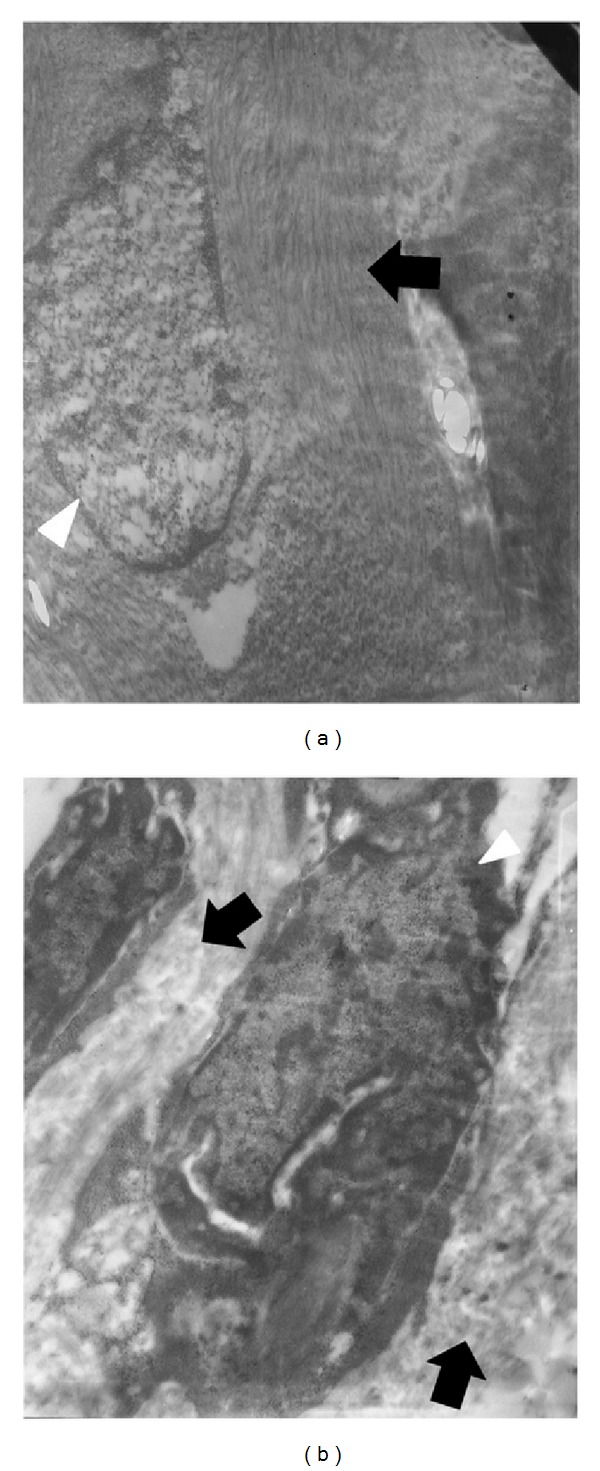
Collagen fibrils in myometrium and fibroids. Comparison of collagen fibril organization in the extracellular matrix of myometrium or uterine fibroid using electron microscopy. (a) Myometrium. Collagen fibrils are tightly packed and well-aligned, as shown by the black arrow. The nucleus is denoted by the white arrowhead. Magnification = 11,500x. (b) Fibroid. The collagen fibrils are randomly aligned and widely spaced, as shown by black arrows. The nucleus is notched and denoted by the white arrowhead. Magnification = 15,500x. Representative sections on samples harvested from a single uterus.

Compared to myometrium, in fibroids not only is the expression of collagen genes increased (as discussed by [71]), but also the amount of mature cross-linked collagen protein is increased and most importantly is altered (discussed in [18]) (Figure 3). The collagen fibrils are shorter and are disordered compared to normal myometrium (discussed by [18, 72]). Furthermore, the ratio of Type I/III collagen is altered. Several investigators demonstrated that Type V collagen, a type thought to be found in fibrotic tissue was a noticeable component of fibroids (as discussed by [73, 74]). Studies have also shown that fibroids and myometrium possess different percentages of the glycosaminoglycans chondroitin sulfate and dermatan sulfate as there is 78% in myometrium and 95% in fibroids (as discussed in [75]). The main glycosaminoglycan is decorin whose presence correlates with fibroid size (as discussed in [76]) and as noted previously interacts with specific regions on the surface of the interstitial collagens and is involved in the organization and assembly of collagen fibrils. Interestingly, decorin exists in fibroids in a higher molecular weight form compared to normal myometrium, as it is glycosylated with longer dermatan sulfate side chains but has unaltered core proteins (as discussed by [55]). These modifications in decorin structure could increase osmotic pressure within the fibroid tissue. Distribution patterns of decorin and collagen were completely different in fibroids compared to normal myometrium when observed by immunofluorescence, and the ratio of decorin to Type I collagen was increased in fibroids (as discussed by [76]). These changes most likely affect the ECM organization of fibroids and would affect its stiffness.

Fibroids have increased fluid content relative to myometrium which could contribute to their mechanical properties and response to certain GnRH analogue therapies (as discussed by [77, 78]). One GnRH analogue, leuprolide acetate, will cause a regression of fibroid size; however, the tumors regrow rapidly after treatment is discontinued (as discussed by [79]). Since fibroids have a low mitotic index this regrowth is thought to be due to changes in the regulation of ECM component of the tumors rather than cell proliferation. A recent study demonstrated that leuprolide acetate may act directly on fibroids by an increase in the ECM genes, COL1A1, fibronectin, and veriscan variant 0, followed by return to normal after five days of treatment (as discussed by [80]). These findings were supported by the presence of GnRH receptors in uterine fibroid and subject-matched myometrium (as discussed by [81]). Similar to the concept of reciprocity for mechanical stress, hydration of the ECM requires an osmotic response regulated by the transcription factor, nuclear factor of activated T cells-5 (NFAT5) that coordinates expression of hyperosmolarity response genes, such as aldose reductase and sodium myoinositol transporter 1 (SMIT), all of which were increased in fibroid cells, compared to myometrial cells (as discussed by [82]). The contribution of hydration to the stiffness of fibroids was supported by viscoelastic measurements of fibroid tumors that took into account the contribution of water to ECM stiffness (as discussed by [2]).

Evidence is accumulating that it is the fibrotic process that contributes to the greater stiffness of fibroids compared to adjacent myometrium (as discussed in [1–3]). Fibrosis was detected by Masson Trichrome staining of collagen in uterine fibroids >1 cm. (as discussed by [83]). Fibrosis was found in 21% of 159 small “seedling” fibroids in premenopausal women and was increased to 50% in the same size tumors in postmenopausal women [84]. Substantial fibrosis, analyzed by morphometry, was noted in 23% of fibroids 2–4 mm in size, while 40% of the 5–9 mm tumors contained fibrotic tissue, a statistically significant increase. Cells were smaller in postmenopausal small fibroids compared to premenopausal fibroids matched within one degree of fibrosis or “fibrous degeneration” (as discussed in [83, 84]). Clearly, cells that could become larger uterine fibroids secreted collagen even before the fibroid was detected clinically. Interestingly, as early as 1983, investigators using electron microscopy observed cells in central regions of fibroids less than 3 mm in size that closely resembled myofibroblasts-cells capable of secreting collagen (as discussed in [85]).

Fibrosis has several components, namely, proliferation and persistence of cells due to resistance of apoptosis and the secretion of collagen by cells and the disposition of abundant highly cross-linked and disoriented collagen fibrils as well as the secretion of proteoglycans and other matrix components (as discussed in [6]). Not only a component of fibrosis is cell proliferation and the formation of new collagen and secretion of proteoglycans, but also an important part of the process is modification of cell proliferation and the correlation of a wide number of genes involved in ECM production including TGF induced myofibroblast differentiation (as discussed in [6]). The stiffness of the ECM that surrounds cells depends on the amount of cross-linking of the newly secreted altered collagen. We have found that LOX is over expressed in fibroids compared to myometrium (unpublished data) which suggests that collagen cross-linking is increased in fibroids. Thus, fibroids meet all of these characteristics of fibrosis. Although some pathologists have traditionally considered fibrosis to be a sign of fibroid senescence, the concept of mechanotransduction challenges this assumption. The changes in the surrounding ECM outlined in the preceding paragraphs would necessarily be accompanied by changes in the mechanical force exerted on resident fibroidal cells, thus leading to changes in cell signaling which would either enhance or inhibit tumor growth, including ECM secretion. Since fibrotic tissue contains abundant collagen, a stiff protein, these findings underscore the role of mechanotransduction in fibroid growth. Even a small amount of increased collagen and other components of the ECM in a cells microenvironment could increase the mechanical force on individual cells and alter cell signaling. Thus, it is not the altered ECM constitution alone but ECM stiffness that has a direct effect on fibroid formation and growth.

Fibrosis is initiated by many triggers which injure a cell, such as extravasation of blood into tissues, oxidative stress, infection, and chronic inflammation. The exact trigger that initiates the fibrotic process in fibroids remains unclear. It is possible that there is not one trigger but several causes, acting either alone, or in concert. For example, resident uterine stem cells might be triggered to undergo altered morphogenesis featuring fibrosis and hypoxia may play a role. The clonality of uterine fibroids (as discussed by [86] and references therein) supports the hypothesis that fibroids arise from a stem-like cell. The nascent fibroid milieu is certainly hypoxic, due in large part to altered angiogenesis, and this observation suggests a role for oxidative stress as a likely trigger for fibroid development and growth (as reviewed in [87]) We and others (as discussed in [88]) have noted that hypoxia might trigger stem cell proliferation giving rise to fibroids. Thus, hypoxia appears to be one underlying pathophysiologic mechanism promoting development of fibroids and a genetic predisposition might be another. Altered expression of Frizzled-related protein 1 was detected on early microarray studies of fibroids (as discussed in [89]), and altered Wingless-type (WNT)/*β*-catenin signaling has recently been reported to promote growth of leiomyoma side-population cells (as discussed in [90]). Thus, current evidence suggests that a specialized myometrial side population of cells (as discussed in [88, 91]) is induced to embark on a fibrotic differentiation, a predilection that is very common. To our thinking, the underlying etiology may be due to the remarkable plasticity and capacity of the uterus to respond to pregnancy-related myometrial growth, with ECM stiffness playing a critical role in expansion of that cell population leading to fibroid growth.

Intriguingly, fibroids are more common than expected in myometrium, immediately adjacent to endometrium compared to other area of the uterus (as discussed in [92]). Thus, it is equally plausible that extravasation of menstrual blood into the myometrium with its accompanying cytokines and growth factors might cause cell injury leading to fibrosis. Consistent with a location-dependent trigger, fibroids are not observed in equal distribution throughout the uterus. Fibroids are more common in the fundus than in the corpus and isthmus and even less common in the cervix as reported by a detailed morphometric study (as discussed in [92]). It has been hypothesized that ECM stiffness may contribute to tumor development in other systems (as discussed in [93, 94]), most likely through the RhoA pathway (as discussed in [95, 96]). Several studies implicate a role for activation of the mammalian target of rapamycin (mTOR) in the pathogenesis of fibroids (as discussed in [97, 98]). Intriguingly, mechanical signaling through P13 K/AKT induces mTOR (as discussed in [99]). Thus, evidence from direct physical measurements, assessment of ECM structural organization, ECM constitution, microscopic analysis, and* in vitro* studies all support the critical role of ECM stiffness in fibroid growth.

## 5. New Directions for Fibroid Treatment

Given that uterine fibroids are primarily composed of an abnormally formed matrix, degradation of the ECM is critical for the resolution of the bulk symptoms caused by these tumors. Thus, the concept of mechanical signaling provides rationale for dissolution of the ECM as a treatment of fibroids. A change in mechanical force and signaling to myofibroblasts within the tumor would thus occur. In theory, the decreased force exerted against the cells would decrease signals that cause matrix deposition and then favor apoptosis of the fibroid cells. Consistent with this tenet, we found that degradation of uterine fibroid collagen by a bacterial (clostridium histolyticum) collagenase significantly reduced the tissue stiffness as measured by rheometry. This collagenase, first isolated over sixty years ago (as described by [100]), only degrades the interstitial collagens and it does not degrade Type IV collagen, the collagen found in blood vessels and nerves (discussed by [101]). Furthermore, this collagenase is inhibited by serum proteins (as discussed by [102]).

The altered mechanical properties of uterine fibroids provide support for the dissolution of ECM as a therapy. Fibroids occur in an environment of increased mechanical stress, but their response to the cues from this environment is paradoxically decreased (as discussed by [1, 2]). Myometrial cells reorient their actin cytoskeleton perpendicular to the axis of applied strain, but fibroid cells do not do so to the same degree (as discussed by [2]). Application of mechanical stress to fibroid cells* in vitro* led to diminished activation of RhoA, in contrast to myometrial cells, suggesting that myofibroblasts have an attenuated response to mechanical cues. Additionally, fibroid tumors exhibited increased stiffness in unconfined compression, compared to adjacent myometrium (discussed in [2]). Treatment of fibroid tissue with purified collagenase derived from clostridium demonstrated collagenolysis and reduced stiffness (as discussed in [3]), confirming the critical role of collagen in the ECM stiffness of fibroids. Likewise, the mechanisms of FUS (or HIFU) and uterine artery embolization (UAE/UFE) in reducing fibroid size are due to necrosis, which also decreases fibroid stiffness by destroying ECM-producing cells, either from heat degradation or coagulation necrosis, respectively.

In keeping with investigations of Vitamin D effects on the ECM in other tissues, a recent study found that Vitamin D inhibits the expression and activities of both MMP2 and MMP9 in fibroid cells (as discussed in [103]). The substrates for MMP2 are numerous and include gelatin; collagen Types I, IV, V, VII, and X; and fibronectin, lamin, aggrecan, tenascin C, and vitronectin, while those of MMP-9 include gelatin; collagen Types IV, V, and XVI; aggrecan; and elastin (as discussed by [104]). Thus low vitamin D serum levels might affect numerous ECM proteins and vitamin D therapy might reduce the fibrotic process in the uterus, similar to the reduction of fibrosis in other tissues. It is very provocative that evidence from epidemiology points to low serum vitamin D as a factor in fibroid formation (as discussed by [105]) and may be one mechanism leading to the differences in fibroid growth in African American compared to Caucasian women (as discussed in [106]). In this study, women with sufficient levels of serum vitamin D had an estimated 32% lower risk of having fibroids.

Uterine fibroids do not grow in a consistently linear pattern, an observation consistent with modulation of growth by an altered ECM. In the same woman, as documented by careful MRI studies, some fibroids grew over six months of time and others were static, while others tended to regress in size. This same clinical investigation reported that women of African American ancestry continued to increase the size of their fibroids after age 35 until menopause. However, women who were identified as white tended to have nongrowing tumors during those ages (as discussed by [107]). When the fibroid tissues of women in this study who underwent surgery were studied for gene expression, dermatopontin was consistently downregulated as reported by others (discussed by [72]). Rho and RAC genes involved in mechanotransduction were elevated in growing tumors. Furthermore it appeared that growing fibroids cells accumulated because there is an absence of cell death signals. These investigators stated that growth of the tumors was also due to the mass accumulation of ECM (discussed by [108]). Recently,* in vitro* studies have shown that fibroid smooth muscle cells grown on different (nonpolymerized versus polymerized) collagen matrices exhibit differences in cell morphology, proliferation, and signaling pathways (as discussed by [109]). Consistent with an increase in ECM stiffness,* in vivo *measurement of fibroid elastography revealed heterogeneity between fibroids and supported an increased stiffness in fibroids in six patients (as discussed by [110]). Collectively, these seminal investigations provide strong evidence for a role of ECM and mechanotransduction in the growth of uterine fibroids. The role of integrins as mechanosensors is important to explore more fully. An important question to be answered is how they are involved in the activation stage of the accumulation of altered ECM (as discussed in [111]). Additional studies are needed to quantify ECM stiffness in growing compared to regressing fibroids as such studies may provide a missing piece to the enigmatic puzzle of fibroid growth.

## 6. Summary

The ECM, especially the interstitial collagens, exert mechanical forces on surrounding cells leading to transmittal of mechanical signals on the cell surface and the initiation of chemical cascades. Uterine fibroids are composed of abundant interstitial collagens (I, III, and V) with fibrils that exhibit a disordered pattern that is increased in amount compared to the adjacent myometrium. Other ECM proteins, especially fibronectin, are also increased relative to myometrium. Proteoglycans especially decorin and versican play roles in the alterations of the ECM organization within fibroids and contribute to the mechanical forces as well and thus to altered mechanical sensing by the cells leading to enhanced accumulation of ECM proteins. The nonlinear growth of fibroids over time and the various patterns of growth and regression of fibroids in the same uterus suggest a local effect of the ECM on mechanical signaling. Therefore, to fully understand uterine fibroids and to elucidate new therapeutic modalities it is essential to investigate further mechanisms of the role the ECM in these common benign tumors.
